# Interictal Single-Voxel Proton Magnetic Resonance Spectroscopy of the Temporal Lobe in Cats with Idiopathic Epilepsy: A Prospective Case–Control Study

**DOI:** 10.3390/vetsci13090848

**Published:** 2026-08-22

**Authors:** Bertuğ Bekir Çiftçi, Gültekin Atalan, Mehmet Ulusan

**Affiliations:** 1 Department of Surgery, Faculty of Veterinary Medicine, Institute of Health Sciences, Erciyes University, 38280 Kayseri, Türkiye; istanbulhayvanhastanesi@gmail.com (B.B.Ç.); gatalan@erciyes.edu.tr (G.A.); 2 Istanbul Animal Hospital, 34779 Istanbul, Türkiye; 3 Department of Internal Medicine, Faculty of Veterinary Medicine, Mehmet Akif Ersoy University, 15030 Burdur, Türkiye

**Keywords:** cat, idiopathic epilepsy, proton magnetic resonance spectroscopy, single-voxel ^1^H-MRS, temporal lobe, N-acetylaspartate, choline, creatine, neuroimaging

## Abstract

***Background:*** Idiopathic epilepsy (IE) is among the most frequent chronic neurological disorders of cats, but by definition it is associated with an unremarkable conventional brain MRI, leaving its temporal lobe pathophysiology poorly characterised. ***Objectives:*** To quantify interictal temporal lobe neurometabolite ratios in cats with IE using bilateral single-voxel ^1^H-MRS and to compare them with healthy controls, and to explore relationships with seizure timing and signalment. ***Methods:*** In this prospective case–control study, 20 client-owned cats with idiopathic epilepsy and 20 healthy control cats underwent bilateral single-voxel ^1^H-MRS (PRESS; TE 135 ms; 10 × 10 × 10 mm voxel centred on the mid-hippocampus and amygdala). N-acetylaspartate (NAA), total choline (tCho), and total creatine (tCr) were quantified and the NAA/tCr, NAA/tCho, tCho/tCr, and tCho/NAA ratios were compared (independent-samples *t*-tests; one-way ANOVA; Spearman correlation; α = 0.05). ***Results:*** Overall, no metabolite ratio differed significantly between groups across both hemispheres, and no ratio correlated with age, sex, breed, coat length, or neutering status. However, the right temporal lobe NAA/tCr ratio was significantly lower in epileptic cats (1.32 ± 0.29) than in controls (1.50 ± 0.20; *p* < 0.05). Seizure timing modulated the left-hemisphere spectrum: left tCho/tCr correlated negatively with the interval since the last seizure (Spearman r = −0.48; *p* = 0.035), and cats with ≤3 days between first and last seizures showed lower left NAA/tCho (0.89 ± 0.23 vs. 1.14 ± 0.24; *p* = 0.032) and higher left tCho/NAA (1.21 ± 0.40 vs. 0.91 ± 0.17; *p* = 0.039) than cats with a longer interval. ***Conclusions:*** Interictal ^1^H-MRS detects temporal lobe metabolic disturbances, including a reduced right temporal lobe NAA/tCr ratio suggesting neuronal or metabolic dysfunction and seizure timing-dependent membrane turnover shifts, in cats with normal-appearing MRI. ^1^H-MRS is therefore a promising non-invasive adjunct for characterising, monitoring, and potentially assisting in the localisation of metabolic abnormalities in feline IE and warrants validation in larger, longitudinally sampled cohorts.

## Simple Summary

Epileptic seizures are among the most common neurological problems in cats, yet in many affected animals, the brain appears entirely normal on conventional magnetic resonance imaging (MRI) and no underlying cause can be identified; these cats are diagnosed with idiopathic epilepsy. Proton magnetic resonance spectroscopy (^1^H-MRS) is a non-invasive technique that measures the biochemical composition of brain tissue rather than its anatomy, enabling microscopic metabolic changes to be detected even when standard images look normal. In this study, we performed ^1^H-MRS of both temporal lobes in 20 cats with idiopathic epilepsy during a seizure-free (interictal) interval and compared them with 20 healthy cats. Although most metabolite values overlapped between the groups, epileptic cats showed a significant reduction in a neuronal or metabolic dysfunction marker in the right temporal lobe, and several metabolite ratios varied according to how recently the cat had seized. These findings indicate that ^1^H-MRS can reveal seizure-related metabolic disturbances that are invisible on routine MRI and may ultimately assist clinicians in diagnosing, lateralising, and monitoring epilepsy in cats.

## 1. Introduction

Recurrent epileptic seizures are the most common chronic neurological presentation in cats, with an estimated prevalence of approximately 2% in referral populations [1,2]. The International Veterinary Epilepsy Task Force (IVETF) recommendations were originally developed for companion animal epilepsy, primarily in dogs, and have subsequently been adopted as a diagnostic framework for feline epilepsy [1,3]. Accordingly, feline epilepsy is commonly classified into structural epilepsy, epilepsy of unknown cause, and idiopathic epilepsy (IE). According to the recommendations of the International Veterinary Epilepsy Task Force (IVETF), IE requires a history of two or more unprovoked seizures at least 24 h apart, seizure onset typically between 6 months and 6 years of age, an unremarkable interictal physical and neurological examination, a normal minimum laboratory database, and an epilepsy-specific brain MRI that is free of clinically relevant abnormalities [1,3]. By definition, therefore, the substrate of feline IE remains invisible to conventional structural imaging, and the disorder is one of exclusion.

Structural epilepsy in cats may frequently involve the temporal lobe. Hippocampal and piriform-lobe necrosis is an uncommon but well-recognised epilepsy-associated syndrome predominantly associated with focal seizure manifestations [4]. Retrospective MRI series have shown that structural epileptogenic lesions commonly involve forebrain structures, including the temporal lobe and limbic system [5,6]. Because epileptic seizures are fundamentally localised to the forebrain, the temporal lobe represents a biologically relevant region for advanced metabolic imaging. Certain anatomical regions affected in feline epilepsy overlap with structures commonly involved in human temporal lobe epilepsy. However, these conditions should not be considered directly analogous. Nevertheless, this anatomical overlap supports investigation of the feline temporal lobe as a target for advanced metabolic imaging biomarkers, even when its macroscopic appearance is normal.

Proton magnetic resonance spectroscopy (^1^H-MRS) extends MRI from anatomy to in vivo biochemistry by resolving tissue resonances of N-acetylaspartate (NAA), choline-containing compounds (tCho), and creatine/phosphocreatine (tCr). NAA is synthesised almost exclusively in neurons and is an accepted surrogate marker of neuronal density, viability, and mitochondrial function; its reduction signals neuronal loss or dysfunction [7,8]. tCho reflects membrane phospholipid turnover and is increased by gliosis, demyelination, and cellular proliferation, whereas tCr, being relatively stable, is widely used as an internal reference, with metabolite ratios (NAA/tCr, NAA/tCho, tCho/tCr) preferred over absolute concentrations in clinical practice [7]. In human mesial TLE, interictal ^1^H-MRS reproducibly demonstrates a reduction in NAA and the NAA/(Cho+Cr) ratio ipsilateral to the seizure focus, attributed to neuronal injury, accompanied by variable increases in Cho and Cr consistent with reactive astrocytosis; these metabolic changes can lateralise the focus even when MRI is unremarkable [9,10,11,12].

In veterinary medicine, ^1^H-MRS remains underexploited. The pivotal study of Olszewska et al. applied bilateral single-voxel temporal lobe ^1^H-MRS to dogs with IE and found no overall difference in metabolite ratios between affected dogs and controls, but identified a significant correlation between the NAA/Cho and Cho/NAA ratios and the time elapsed since the last seizure—implying a dynamic, reversible interictal metabolic signature rather than a fixed deficit [13]. Subsequent canine work extended ^1^H-MRS to the thalamus, where antiepileptic drug-treated epileptic dogs showed reduced NAA/Cr, underscoring both regional and treatment-related metabolite variation [14,15,16]. To date, however, no evidence-based ^1^H-MRS study of the temporal lobe has been reported in cats with IE, and the interictal neurometabolic profile of the feline temporal lobe is entirely uncharacterised.

We therefore conducted, to our knowledge, the first bilateral single-voxel ^1^H-MRS study of the temporal lobe in cats with IE during the interictal period. We hypothesised that, despite normal-appearing MRI, epileptic cats would exhibit measurable temporal lobe metabolite-ratio differences relative to healthy controls—particularly a reduction in NAA-based ratios indicative of neuronal compromise—and that these differences would be modulated by the temporal relationship between imaging and the most recent seizure. The specific objectives were: (i) to quantify and compare interictal NAA/tCr, NAA/tCho, tCho/tCr, and tCho/NAA ratios in both temporal lobes of cats with IE and healthy controls; (ii) to test whether these ratios are associated with signalment (age, sex, breed, coat length, neutering status); and (iii) to examine their relationship with seizure timing.

## 2. Materials and Methods

### 2.1. Study Design, Ethics, and Reporting

This was a prospective, single-centre, case–control study conducted between July 2022 and March 2023 at the Istanbul Animal Hospital (Istanbul, Türkiye). On 7 September 2022, the Erciyes University Animal Experiments Local Ethics Committee (HADYEK) unanimously determined that the study falls within the scope of “clinical applications for diagnostic and treatment purposes” as specified in Article 8, paragraph (K), item 1 of the HADYEK Directive and is therefore exempt from HADYEK approval. The study was supported by the Erciyes University Scientific Research Projects Unit (Project No. TDK-2023-13073). Written informed consent was obtained from all owners before enrolment, including specific consent for general anaesthesia and, where performed, cerebrospinal fluid (CSF) sampling. The manuscript is reported in accordance with the ARRIVE 2.0 guidelines for animal research [15].

### 2.2. Animals, Inclusion, and Exclusion Criteria

Forty client-owned cats were enrolled in two groups. The IE group comprised 20 cats presented for recurrent epileptic seizures [1]. The diagnosis of idiopathic epilepsy was established following a standardised diagnostic work-up based on the recommendations of the International Veterinary Epilepsy Task Force (IVETF), including clinical history, interictal neurological examination, minimum database evaluation, and epilepsy-specific MRI. Cats fulfilled the study inclusion criteria, including two or more unprovoked seizures ≥24 h apart, a normal interictal neurological examination, no history of trauma or relevant prior disease, no prior antiepileptic treatment, and unremarkable haematology, serum biochemistry, venous blood gas/electrolyte analysis, and 1.5-T brain MRI. The control group comprised 20 cats without any history of seizures or neurological disease that were presented for thoracic or lumbar spinal imaging for unrelated clinical reasons and underwent cranial MRI and ^1^H-MRS after owner consent. Cats were excluded if structural intracranial abnormalities were detected on MRI, if any reactive or metabolic cause of seizures was identified, or if image quality was inadequate for spectral analysis. All cats included in the idiopathic epilepsy group were treatment-naive at the time of MRI and MRS acquisition. Previous antiseizure medication was an exclusion criterion for study enrolment.

The 40 cats represented five breeds (Domestic Shorthair/tabby, Domestic Longhair/ginger, Scottish Fold, British Shorthair, and Russian Blue). In the IE group, the median age range spanned 8 months to 6 years; 12 of 20 cats were female (9 spayed) and 8 were male (all neutered). Signalment and seizure history for the IE group are summarised in Table 1. In the IE group, owner-recorded video and history indicated unilateral-onset focal seizures with orofacial automatisms (facial twitching, hypersalivation, lip-smacking, chewing, and licking) in all 20 cats, with secondary generalisation in 8; the ictal period lasted 40 s to 3 min and the mean post-ictal period was approximately 15 min.

### 2.3. Clinical, Laboratory, and Electrodiagnostic Evaluation

All cats underwent a standardised general and neurological examination (mental status, posture, gait, postural reactions, proprioception, hopping, menace response, and olfaction) recorded on a structured case form. Haematology (IDEXX ProCyte), serum biochemistry including ammonia and total thyroxine (IDEXX Catalyst One, IDEXX Laboratories, Westbrook, ME, USA), and venous blood gas electrolytes (IDEXX VetStat, IDEXX Laboratories, Westbrook, ME, USA) were performed in all cats. CSF was obtained from the atlanto-occipital cisterna in 5 IE cats for which owners consented and was analysed cytologically and biochemically. Electroencephalography (EB Neuro NEMUS, EB Neuro S.p.A., Florence, Italy; 12 subdermal stainless-steel electrodes) was recorded for 14–29 min under medetomidine sedation (40 µg/kg IV) in a darkened, quiet room.

### 2.4. Anaesthesia

After clinical and laboratory evaluation, each cat was sedated with medetomidine hydrochloride (40–80 µg/kg IV) and, 10 min later, induced with ketamine (1 mg/kg IV) for general anaesthesia. Following imaging, sedation was reversed with atipamezole (0.1 mg/kg IM); mean recovery time was 10 min.

### 2.5. MRI and ^1^H-MRS Acquisition Protocol

Imaging was performed on a 1.5-Tesla scanner (Siemens Magnetom Sempra, Siemens Healthineers, Malvern, PA, USA) with an 8-channel Ultraflex small coil, with each anaesthetised cat positioned in dorsal recumbency. The structural protocol comprised transverse T1, T2, T2-FLAIR, T2*-GRE, diffusion-weighted, and post-contrast T1 sequences, with sagittal T2/T1 and coronal T1 sequences; gadobutrol (0.1 mmol/kg IV) was used for contrast. Mean structural MRI time was approximately 30 min, and all brain parenchyma and lobes were assessed for pathological signal or enhancement.

**Figure 1:**
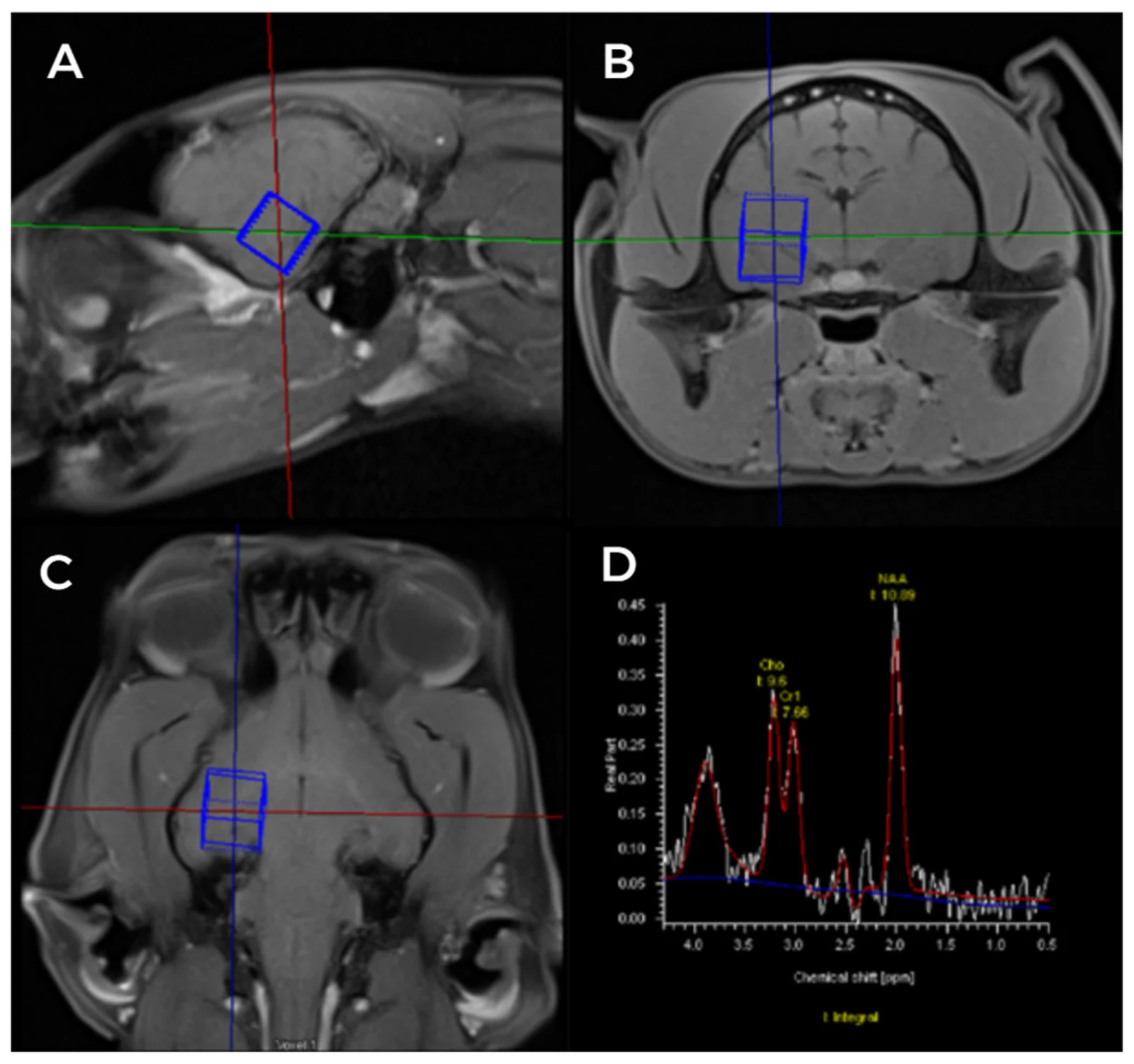
Voxel placement in the temporal lobe, including the hippocampus and part of the amygdala, on post-contrast T1-weighted MR images in the (**A**) sagittal, (**B**) transverse, and (**C**) dorsal planes. (**D**) ^1^H-MRS spectrum.

In cats without structural abnormalities, bilateral single-voxel ^1^H-MRS was performed using PRESS spin-echo localisation (TR 1500 ms; TE 135 ms; ~256 averages). Three CHESS water-suppression cycles (25.6 ms Gaussian RF pulses) preceded localisation. A cubic 10 × 10 × 10 mm voxel of interest was positioned bilaterally in the right and left temporal lobe regions to incorporate the middle portion of the hippocampus and the amygdala (Figure 1). Spectral post-processing (baseline correction, noise filtering, peak calibration, and Fourier transformation) was performed with an automated, operator-independent system (Spectro, Syngo XA12, Siemens Healthineers, Malvern, PA, USA). The metabolite peaks were assigned at 2.02 ppm (NAA), 3.22 ppm (tCho), and 3.02 ppm (tCr), and the NAA/tCr, tCho/tCr, NAA/tCho, and tCho/NAA ratios were computed automatically from the resonance areas. Mean acquisition time was approximately 6 min 32 s per hemisphere (~13 min 4 s per cat). All examinations were acquired under general anaesthesia using the same coil, by the same operator, and quantifying the same metabolites, to minimise inter-observer and technical variability.

### 2.6. Statistical Analysis

Data were analysed in SPSS v24 (IBM Corp., Armonk, NY, USA). Descriptive statistics (mean, standard deviation, minimum, maximum) were calculated for all peak-area ratios. Normality was assessed with the Shapiro–Wilk test (*n* < 50); data were normally distributed (*p* > 0.05), supporting parametric analysis. Between-group comparisons (IE vs. control; and by sex, coat length, age band, and neutering status) used independent-samples *t*-tests, and comparisons across more than two groups (breed) used one-way ANOVA. Associations between metabolite ratios and the time since the first and last seizures were examined with Spearman correlation and with single-factor repeated-measures ANCOVA per voxel side; logistic regression explored predictor effects. Statistical significance was set at α = 0.05 (two-tailed). For the principal between-group comparison, the mean difference, 95% confidence interval, and Cohen’s d effect size are reported.

## 3. Results

### 3.1. Clinical, Laboratory, Electrodiagnostic, and MRI Findings

**Figure 2:**
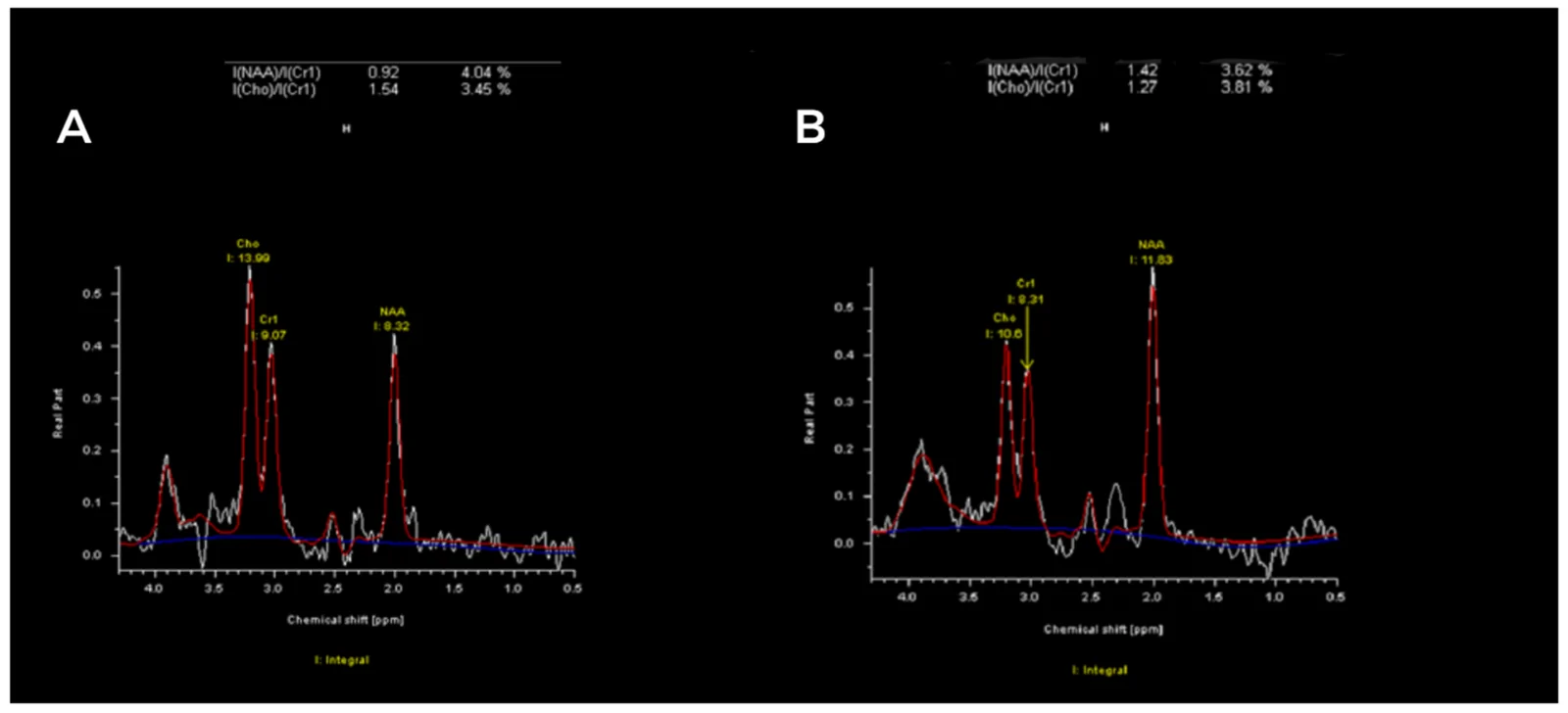
Single-voxel spectrum acquired at the level of the temporal lobe showing metabolite peaks of N-acetylaspartate (NAA) at 2.0 ppm, creatine (Cr) at 3.0 ppm, choline (Cho) at 3.2 ppm and the calculated metabolite ratios in a mixed-breed dog with degenerative disc disease included in the control group (**A**) and in a 5-year-old mixed-breed male dog with idiopathic epilepsy and last seizure 7 days ago (**B**).

Neurological examinations were unremarkable in all 40 cats. Haematology, serum biochemistry (including ammonia and total thyroxine), and venous blood gas electrolytes were within reference intervals in every cat. CSF analysis in the 5 sampled IE cats was normal (e.g., total nucleated cell count ≤ 5 cells/µL, total protein 18 mg/dL, negative culture), with no evidence of inflammatory, infectious, or neoplastic disease. Under medetomidine sedation, EEG in both groups showed the expected high-amplitude slow-wave (delta 0.5–4 Hz, theta 4–7 Hz) activity, symmetric across hemispheres, with no epileptiform discharges, spikes, sharp waves, or focal/generalised abnormalities. Structural brain MRI was normal in all IE and control cats, with no mass effect, midline shift, or pathological contrast enhancement. In the IE group, the first seizure had occurred a mean of 15.6 days (range 1–180 days) before imaging, and the most recent seizure a mean of 24.05 h (range 1–96 h) before imaging (Figure 2).

### 3.2. Bilateral Temporal Lobe ^1^H-MRS: Raw Metabolites

Mean interictal NAA, tCho, and tCr peak values for both temporal lobes are presented in Table 2. Within each group, right and left hemispheric values did not differ significantly for any metabolite (*p* > 0.05), and the spectral pattern was qualitatively similar in epileptic and healthy cats.

### 3.3. Metabolite Ratios: Epileptic Cats Versus Controls

Metabolite ratios for both hemispheres are compared between groups in Table 3. Across the four ratios and both hemispheres, the single statistically significant between-group difference was a reduction in the right temporal lobe NAA/tCr ratio in epileptic cats (1.32 ± 0.29) relative to controls (1.50 ± 0.20; mean difference −0.18, 95% CI −0.35 to −0.02; t = −2.24, *p* = 0.031; Cohen’s d ≈ 0.72), consistent with reduced ipsilateral neuronal or metabolic dysfunction. The left-hemisphere NAA/tCr ratio showed the same downward trend without reaching significance (1.26 ± 0.31 vs. 1.41 ± 0.24; *p* = 0.079), as did the left tCho/NAA ratio (*p* = 0.067). No other ratio differed between groups.

### 3.4. Influence of Signalment

Metabolite ratios were not significantly associated with breed (one-way ANOVA, all *p* > 0.05), coat length, age band (≤24 vs. >24 months), sex, or neutering status (independent-samples *t*-tests, all *p* > 0.05) in either hemisphere. The interictal temporal lobe spectrum therefore appeared robust to the demographic heterogeneity of the cohort.

### 3.5. Influence of Seizure Timing

Seizure timing exerted a measurable, lateralised effect on the left temporal lobe spectrum (Table 4). The left tCho/tCr ratio correlated negatively and moderately with the interval since the most recent seizure (Spearman r = −0.48; *p* = 0.035): the shorter that interval, the higher the left tCho/tCr ratio. When cats were dichotomised by the interval between their first and last recorded seizures, those with ≤3 days between seizures had significantly lower left NAA/tCho (0.89 ± 0.23 vs. 1.14 ± 0.24; t = −2.38, *p* = 0.032) and significantly higher left tCho/NAA (1.21 ± 0.40 vs. 0.91 ± 0.17; t = 2.22, *p* = 0.039) than cats with a longer inter-seizure interval. No metabolite ratio correlated significantly with the time since the first seizure (all *p* > 0.05).

## 4. Discussion

This study provides, to our knowledge, the first interictal single-voxel ^1^H-MRS characterisation of the temporal lobe in cats with idiopathic epilepsy. Three findings stand out. First, despite normal-appearing structural MRI, epileptic cats exhibited a significant reduction in the right temporal lobe NAA/tCr ratio relative to controls, with a medium-to-large effect size. Second, the global between-group spectrum was otherwise preserved, mirroring the canine experience. Third, the left temporal lobe spectrum varied systematically with seizure timing, indicating a dynamic rather than fixed interictal metabolic disturbance.

Because NAA is an established neuronal and mitochondrial marker, a lower NAA/tCr ratio is conventionally interpreted as reflecting neuronal or metabolic dysfunction rather than being specific evidence of neuronal loss [7,8]. The right-sided NAA/tCr reduction we observed is broadly consistent with the hallmark NAA decline in human mesial TLE, where interictal ^1^H-MRS detects neuronal compromise ipsilateral to the focus even when MRI is normal, and contributes to non-invasive lateralisation [9,10,11,12]. That a comparable signal is detectable in feline IE—a disorder defined by unremarkable MRI—supports the central hypothesis that microscopic, spectroscopically visible temporal lobe pathology underlies at least some cases currently classified as idiopathic, and that ^1^H-MRS may eventually help localise a putative epileptogenic focus.

Our overall negative between-group comparison is concordant with Olszewska et al., who found no global temporal lobe metabolite ratio differences between epileptic and control dogs when seizure timing was ignored [13]. Crucially, that study also demonstrated that NAA/Cho and Cho/NAA ratios correlated with the time since the last seizure, interpreted as a transient, partly reversible post-ictal metabolic shift. Our feline data reproduce and extend this phenomenon: left NAA/tCho was lower and left tCho/NAA higher in cats imaged closer to seizure activity, and left tCho/tCr declined as the interval since the last seizure lengthened. The convergence of choline-based ratios in both species points to seizure-associated membrane phospholipid turnover and reactive glial change—the same biology proposed for the Cho elevations of human TLE [9]—that wax and wane around the ictus. This timing dependence is clinically important: it implies that the diagnostic yield of ^1^H-MRS in feline epilepsy depends on when, relative to the last seizure, the cat is scanned, and that imaging time should be standardised and recorded in future studies and in clinical interpretation.

The lateralisation of our findings, neuronal-integrity loss expressed on the right and timing-dependent membrane changes on the left, should be interpreted cautiously given the sample size, but is biologically plausible: focal feline epilepsies, including hippocampal necrosis syndromes, are frequently unilateral in onset [4,6,17,18], and all cats in this cohort had unilateral-onset focal seizures. Asymmetric temporal lobe metabolism is also typical of human TLE. The complementary thalamic canine study, in which antiepileptic drug-treated dogs showed reduced NAA/Cr, further indicates that metabolite ratios vary by region and by treatment status [14,19,20]. Importantly, all cats included in the present study were treatment-naive at the time of MRI and MRS acquisition. The absence of antiseizure medication before imaging minimised the potential confounding effects of drug-induced alterations in cerebral metabolite concentrations and therefore represents a methodological strength of the present study. Consequently, the observed metabolic alterations are more likely attributable to the epileptic process itself rather than to medication.

The absence of any association between metabolite ratios and age, sex, breed, coat length, or neutering status is reassuring for the biomarker’s potential robustness, suggesting that a single interictal temporal lobe spectrum is not strongly confounded by signalment within the studied range. It also argues that the right NAA/tCr reduction reflects disease rather than demographic structure, although larger cohorts are needed to exclude small confounding effects and to derive species- and field-strength-specific reference intervals.

In the present study, metabolite ratios normalised to total creatine (tCr) were used because this approach is widely applied in both clinical and veterinary proton MRS and reduces variability associated with absolute metabolite quantification. However, although tCr is generally considered relatively stable, creatine metabolism may not remain completely constant under all pathological conditions, including epilepsy. Therefore, absolute metabolite quantification may provide complementary information in future investigations. Because identical acquisition protocols were applied consistently across both study groups, the use of tCr normalisation was considered appropriate for the comparative analyses performed in the present study.

### 4.1. Clinical Implications

^1^H-MRS is non-invasive, can be appended to a standard epilepsy MRI protocol in roughly 13 min, and requires no additional contrast. Our data suggest three potential clinical roles in feline epilepsy: detection of microscopic temporal lobe pathology that is occult on structural MRI; lateralisation of a candidate focus via asymmetric NAA-based ratios; and monitoring of the interictal/post-ictal metabolic state through choline-based ratios indexed to seizure timing. Realising these roles will require standardised acquisition, reference data, and prospective correlation with seizure frequency and treatment response.

### 4.2. Limitations and Future Directions

Several limitations should be considered when interpreting the present findings. First, the sample size (20 cats with idiopathic epilepsy and 20 control cats) was relatively modest, limiting statistical power for subgroup analyses and precluding correction for multiple comparisons. Consequently, the single significant between-group difference should be regarded as hypothesis-generating and requires confirmation in larger, ideally multicenter studies. Second, the cross-sectional case–control design provided only a single interictal examination for each cat. Because imaging was performed at variable intervals after the most recent seizure, the observed metabolic alterations cannot be interpreted as reflecting longitudinal changes over the course of the disease. Another limitation is that quantitative seizure-frequency data, including monthly seizure frequency, cumulative seizure number, and occurrence of cluster seizures, were not prospectively collected. Therefore, potential associations between seizure burden and cerebral metabolite alterations could not be evaluated. Future longitudinal studies incorporating standardised seizure-frequency metrics together with serial MRS examinations may provide further insight into the relationship between epilepsy severity and metabolic changes. Cerebrospinal fluid examination was performed in only five cats because the procedure required additional owner consent. Although all enrolled cats underwent comprehensive clinical, neurological, laboratory, and epilepsy-specific MRI evaluations without evidence of structural, inflammatory, infectious, metabolic, or toxic disease, inflammatory disorders cannot be completely excluded in cats that did not undergo CSF analysis. This limitation should be considered when interpreting the findings of the present study. Methodologically, metabolite quantification was based on metabolite ratios rather than absolute concentrations, and additional metabolites such as glutamate-glutamine, myo-inositol, and lactate were not evaluated. Furthermore, histopathological confirmation was not available. Although normalisation to total creatine is widely accepted in clinical and veterinary proton MRS, future studies incorporating absolute metabolite quantification and a broader metabolite profile may provide complementary information regarding epileptic brain metabolism. Finally, all examinations were performed under an identical anaesthetic protocol using the same drugs, dosages, imaging system, and acquisition parameters. Although medetomidine and ketamine may influence cerebral metabolism and spectroscopic measurements, these potential effects were expected to be equally distributed between the study groups, thereby minimising systematic bias. Nevertheless, the influence of anaesthesia on brain metabolite measurements should be considered when interpreting the present findings and warrants further investigation.

Future studies should therefore include larger, better-matched cohorts, standardised longitudinal follow-up, serial MRS acquisitions performed at predefined intervals after seizures, absolute metabolite quantification, multi-voxel or chemical-shift imaging, and correlation with EEG findings, treatment response, and, where available, neuropathological data. Such studies will be important for establishing the diagnostic and prognostic value of temporal lobe ^1^H-MRS in feline idiopathic epilepsy and for determining its potential role in clinical practice.

## 5. Conclusions

Interictal single-voxel ^1^H-MRS of the temporal lobe is feasible in cats and reveals metabolic disturbances that conventional MRI cannot: a significant reduction in the right temporal lobe NAA/tCr ratio, suggesting neuronal or metabolic dysfunction, together with seizure timing-dependent changes in left-hemisphere choline-based ratios. The observed right-sided metabolic alteration should be interpreted as an exploratory hemispheric association rather than definitive evidence of epileptogenic focus lateralisation. Although most metabolite ratios did not differ between epileptic and healthy cats and the findings are limited by cohort size, these results establish that spectroscopically detectable, lateralised, and time-dependent temporal lobe pathophysiology exists in feline idiopathic epilepsy. ^1^H-MRS is thus a promising non-invasive adjunct for characterising, monitoring, and potentially assisting in the localisation of metabolic abnormalities in feline epilepsy, and merits validation in larger, longitudinally sampled, time-standardised studies before routine clinical adoption.

## Figures and Tables

**Table 1:** Signalment and seizure history of the 20 cats with idiopathic epilepsy.

Case	Age	Breed	Sex	Coat	Neuter	First sz (d Ago)	Last sz (h Ago)
E1	6 y	Tabby	F	Short	Spayed	20	24
E2	8 m	Tabby	F	Long	Intact	7	20
E3	4 y	Tabby	F	Short	Spayed	1	10
E4	3 y	Scottish Fold	F	Long	Spayed	6	5
E5	7 m	Ginger	M	Short	Intact	3	20
E6	3 y	Ginger	F	Short	Intact	2	1
E7	6 y	Ginger	M	Short	Neutered	3	2
E8	2 y	British SH	F	Short	Spayed	18	48
E9	6 y	Tabby	M	Short	Neutered	1	18
E10	6 y	Tabby	F	Short	Spayed	2	16
E11	4 y	Tabby	F	Short	Spayed	180	72
E12	1.5 y	Tabby	F	Short	Spayed	7	24
E13	6 y	Tabby	M	Short	Neutered	21	96
E14	9 m	Ginger	M	Long	Neutered	12	8
E15	3 y	Tabby	F	Short	Spayed	4	20
E16	5 y	Tabby	F	Short	Spayed	14	48
E17	7 m	Tabby	F	Short	Intact	2	1
E18	5 y	Russian Blue	M	Short	Neutered	3	16
E19	6 y	Tabby	M	Short	Neutered	4	24
E20	1 y	Tabby	M	Short	Neutered	2	8

*F, female; M, male; SH, Shorthair; sz, seizure; d, days; h, hours. First sz = days before imaging that the first seizure occurred; Last sz = hours before imaging of the most recent seizure. The variables “First sz” and “Last sz” represent the time elapsed before MRI examination and do not indicate the interval between consecutive seizure events.*

**Table 2:** Mean (±SD) interictal single-voxel ^1^H-MRS peak values (arbitrary units, ppm scale) of the right and left temporal lobes in cats with idiopathic epilepsy (IE; *n* = 20) and healthy controls (*n* = 20).

Metabolite (ppm)	IE Right	IE Left	Control Right	Control Left
NAA	10.97 ± 1.82	10.13 ± 2.24	11.45 ± 1.94	11.04 ± 1.60
tCho	11.56 ± 2.51	10.51 ± 3.18	11.22 ± 1.64	10.23 ± 1.78
tCr	8.47 ± 1.33	8.22 ± 1.36	7.74 ± 1.31	8.10 ± 1.77

*NAA, N-acetylaspartate; tCho, total choline; tCr, total creatine. No within-group right-vs-left difference reached significance (independent-samples t-test, p > 0.05).*

**Table 3:** Interictal temporal lobe ^1^H-MRS metabolite ratios (mean ± SD) in cats with idiopathic epilepsy versus healthy controls (independent-samples *t*-test).

Ratio/Hemisphere	IE (*n* = 20)	Control (*n* = 20)	t	*p*
NAA/tCr—right	1.32 ± 0.29	1.50 ± 0.20	−2.24	0.031 *
tCho/tCr—right	1.37 ± 0.24	1.47 ± 0.21	−1.34	0.190
NAA/tCho—right	0.98 ± 0.22	1.04 ± 0.17	−0.92	0.366
tCho/NAA—right	1.07 ± 0.26	1.00 ± 0.25	0.89	0.380
NAA/tCr—left	1.26 ± 0.31	1.41 ± 0.24	−1.80	0.079
tCho/tCr—left	1.31 ± 0.43	1.28 ± 0.18	0.32	0.748
NAA/tCho—left	1.01 ± 0.26	1.11 ± 0.12	−1.44	0.159
tCho/NAA—left	1.06 ± 0.34	0.91 ± 0.11	1.88	0.067

* p < 0.05. Values recomputed from per-cat peak areas; the right NAA/tCr reduction is the single significant between-group difference.

**Table 4:** Seizure timing-dependent changes in left temporal lobe ^1^H-MRS ratios in cats with idiopathic epilepsy (Spearman correlation and independent-samples *t*-test).

Analysis (Left Hemisphere)	Shorter Interval	Longer Interval	Statistic	*p*
tCho/tCr vs. time since last seizure	—	—	r = −0.48	0.035 *
NAA/tCho (≤3 d vs. >3 d first–last)	0.89 ± 0.23	1.14 ± 0.24	t = −2.38	0.032 *
tCho/NAA (≤3 d vs. >3 d first–last)	1.21 ± 0.40	0.91 ± 0.17	t = 2.22	0.039 *

* p < 0.05. “Shorter/longer interval” denotes ≤3 vs. >3 days between the first and last recorded seizures for the dichotomised analyses.

## Data Availability

The original contributions presented in this study are included in the article. Further inquiries can be directed to the corresponding author.

## Abbreviations

The following abbreviations are used in this manuscript:

1H-MRS	Proton magnetic resonance spectroscopy
MRI	Magnetic resonance imaging
IE	Idiopathic epilepsy
IVETF	International Veterinary Epilepsy Task Force
TLE	Temporal lobe epilepsy
NAA	N-acetylaspartate
tCho	Total choline (choline-containing compounds)
tCr	Total creatine (creatine + phosphocreatine)
PRESS	Point-RESolved Spectroscopy
CHESS	Chemical-shift-selective (water suppression)
VOI	Volume of interest
TR	Repetition time
TE	Echo time
ppm	Parts per million
T	Tesla
FLAIR	Fluid-attenuated inversion recovery
GRE	Gradient-recalled echo
CSF	Cerebrospinal fluid
EEG	Electroencephalography
IV	Intravenous
IM	Intramuscular
ANOVA	Analysis of variance
ANCOVA	Analysis of covariance
SD	Standard deviation
CI	Confidence interval
ARRIVE	Animal Research: Reporting of In Vivo Experiments
